# Isoliquiritigenin attenuates neuroinflammation in mice model of Parkinson’s disease by promoting Nrf2/NQO-1 pathway

**DOI:** 10.1515/tnsci-2022-0239

**Published:** 2022-09-12

**Authors:** Lijuan Huang, Yan Han, Qingmin Zhou, Zhihao Sun, Jianhui Yan

**Affiliations:** Nursing College of Xiangnan University, Affiliated Hospital of Xiangnan University, Chenzhou 423000, Hunan Province, China; Department of General Practice, Affiliated Hospital of Xiangnan University, Chenzhou 423000, Hunan Province, China; Clinical College of Xiangnan University, Affiliated Hospital of Xiangnan University, Chenzhou 423000, Hunan Province, China; Department of Gastroenterology, Affiliated Hospital of Xiangnan University, Chenzhou 423000, Hunan Province, China

**Keywords:** Parkinson’s disease, isoliquiritigenin, neuroinflammation, Nrf2

## Abstract

Parkinson’s disease (PD) is a common neurodegenerative disease that severely affects the quality of life of patients. There is no specific drug for PD up to now. Previous studies have shown that neuroinflammation plays an important role in the pathogenesis of PD. Isoliquiritigenin (ILG) is thought to have a variety of biological activities including anti-inflammatory. However, to date, no studies have reported the role of ILG on neuroinflammation in PD *in vivo*. This study aimed to investigate the effect of ILG on PD *in vivo* and its mechanism, and to provide an experimental basis for clinical treatment of PD. Our results showed that ILG at a concentration of 20 mg/kg was effective in reducing the number of rotations in PD mice. In addition, ILG increased the expression of tyrosine hydroxylase and decreased the expression of α-synuclein. The results also showed that ILG reduced the expression of Iba1, IL-1β, IL-6, and TNF-α. Not only that, ILG also upregulated the expression of Nrf2 and NQO-1 *in vivo*. Our results suggest that ILG significantly attenuates neurological deficits in PD, and the mechanism may be through the activation of the Nrf2/NQO-1 signaling pathway to reduce neuroinflammation. Moreover, our findings provide a new therapeutic strategy for PD.

## 1 Introduction

Parkinson’s disease (PD) is the second most common neurodegenerative disease in the world, accounting for about 2% of the population over 60 years of age, with the acceleration of the aging of the world population, the incidence of PD is gradually increasing [1,2]. PD is a functional disease characterized by progressive loss of dopaminergic neurons in the substantia nigra (SN) and accumulation of Lewy bodies in the brain, and its clinical manifestations are mainly motor dysfunction [3,4]. Although the exact pathogenesis of PD is not yet fully elucidated, evidence from clinical and preclinical studies suggests that neuroinflammation and oxidative stress may play a pivotal role in the pathogenesis of PD [5–7]. Among them, a number of clinical and experimental studies suggest that the relationship between the activation of microglia and neuroinflammation may be a vital regulator of the loss of dopaminergic neurons in PD [8,9]. Despite the efforts of researchers worldwide, there are still no specific therapeutic drugs to terminate the progression of PD; in addition, clinical trials of gene therapy for PD have failed to prevent or slow the progression of PD [10–14]. Therefore, there is an urgent need to find new drugs to prevent the loss of dopaminergic neurons in patients with PD.

Isoliquiritigenin (ILG) is a component of *Glycyrrhiza uralensis* (*G. uralensis*) with a variety of biological activities [15]. Previous research evidence revealed that ILG has shown positive effects in the treatment of human diseases, including anti-tumor effects, hepatoprotective effects, and cardioprotective effects [15]. It has been shown that ILG can inhibit the production of tumor necrosis factor-α (TNF-α) and interleukin-6 (IL-6) induced by lipopolysaccharide *in vitro* [16]. In addition, it has been recently reported that intraperitoneal injection of ILG attenuates brain injury and neurological deficits in a model of intracerebral hemorrhage, and that this effect may be achieved through the Nrf2 signaling pathway [17]. Furthermore, Liu et al. demonstrated that ILG reduced neuroinflammation and improved neurological function in traumatic brain injury (TBI) rats [18]. Moreover, ILG was shown to protect and attenuate oxidative stress after TBI via the Nrf2–ARE signaling pathway [19]. In addition, some studies have shown that ILG is an effective hMAO inhibitor, which has competitive inhibition on hMAO-A and mixed inhibition on hMAO-B. ILG has multi-target properties, suitable pharmacokinetic prediction, and toxicity distribution, which makes ILG a potential flavonoid for the treatment of PD and its related neurological symptoms [20,21].

The above studies indicate that ILG and Nrf2-associated signaling pathways are closely related. Most importantly, ILG was shown to attenuate 6-hydroxydopamine (6-OHDA)-induced motor dysfunction and prevent apoptosis of dopaminergic neurons in PD [22]. However, the specific molecular mechanisms have not been fully elucidated. The purpose of this study was to investigate the effect of ILG on neuroinflammation and its possible molecular mechanisms in the mice model of PD.

## 2 Materials and methods

### 2.1 Animals

Male C57BL/6J mice, weighing between 20 and 25 g (8–10 weeks), were obtained from Charles River Laboratories. All mice were housed in a specific pathogen-free environment with controlled light, temperature, and humidity (12 h light/dark cycle, constant temperature of approximately 25°C, and relative humidity of about 55%). All mice had free access to standard food and water for the duration of the experiment.


**Ethical approval:** The research related to animals’ use has been complied with all the relevant national regulations and institutional policies for the care and use of animals. All experimental procedures and animal care were approved by the Laboratory Animal Ethics Committee of Xiangnan University and were performed according to the guidelines of the National Institutes of Health on the care and use of animals (Ethical Approval Number 2021086).

### 2.2 PD model

We used 6-OHDA to establish a PD model. Briefly, 1% pentobarbital sodium 50 mg/kg was used to anesthetize the mice and the mice were immobilized on a stereotactic frame (RWD, 68001, China). 6-OHDA solution (Sigma-Aldrich, 3 µL, 5 mg/mL in sterile saline containing 0.02% ascorbic acid) was injected into the right substantia nigra pars compacta (SNc) by utilizing microliter syringe at an infusion rate of 0.5 µL/min with a Hamilton syringe and back pump (RWD). Set bregma as the coordinate origin and locate the body surface projection of the injection site according to the following reference coordinates: anteroposterior, −3 mm, mediolateral, +1.3 mm, and dorsoventral, −4.7 mm. After waiting for 5 min, the needle was slowly withdrawn.

### 2.3 Administration of 6-OHDA and ILG

During the preparation of PD model, the total dose of 6-OHDA injected into each animal was 15 µg, and the injection time was maintained for 2 min. Moreover, according to the pre-experiments of this study and the results of Zeng et al. [17] the optimal ILG intraperitoneal dose was 20 mg/kg. After the successful establishment of PD model, the mice were injected intraperitoneally for ILG 14 days continuously.

### 2.4 Rotation behavior assessment

Apomorphine hydrochloride (APO; Sigma-Aldrich) was dissolved in sterile saline containing 0.02% ascorbic acid and subcutaneously administered at a dose of 0.5 mg/kg of body weight. The mice were injected with APO (0.5 mg/kg) subcutaneously, and placed in square chamber (40 cm^2^), and the number of contralateral turns within a period (30 min) was recorded. Mice with more than seven contralateral turns per minute were used as valid PD model.

### 2.5 Quantitative real-time polymerase chain reaction (qRT-PCR)

We extracted the total RAN in the fourth week after the success of the PD model. Total RNA was extracted from mouse mesencephalon tissue using TRIzol reagent (Biosharp, China) according to the manufacturer’s instructions. Reverse transcription was conducted with the PrimeScript RT reagent kit (Accurate Biology). PCR was performed by using a 7500 Real-Time PCR system (Applied Biosystems, Foster City, CA, USA). Each sample was analyzed in triplicate. The relative gene expression levels reported in this study were analyzed with the 2^−ΔΔ^ Ct method. The primers used to measure mRNA expression levels are shown in Table 1.

**Table 1:** Primer sequences for qRT-PCR

Gene	Primer sequence (5′ to 3′)
M-GAPDH	F:GGTGAAGGTCGGTGTGAACG
	R:CTCGCTCCTGGAAGATGGTG
M-Iba-1	F:CTTGAAGCGAATGCTGGAGAA
	R:GGCAGCTCGGAGATAGCTTT
M-IL-β	F:GAAATGCCACCTTTTGACAGTG
	R:TGGATGCTCTCATCAGGACAG
M-IL-6	F:CTGCAAGAGACTTCCATCCAG
	R:AGTGGTATAGACAGGTCTGTTGG
M-TNF-α	F:CTGAACTTCGGGGTGATCGG
	R:GGCTTGTCACTCGAATTTTGAGA
M-Nrf2	F:TAGATGACCATGAGTCGCTTGC
	R:TAGATGACCATGAGTCGCTTGC
M-NQO-1	F:AGGATGGGAGGTACTCGAATC
	R:TGCTAGAGATGACTCGGAAGG
M-TH	F:CCAAGGTTCATTGGACGGC
	R:CTCTCCTCGAATACCACAGCC

### 2.6 Western blot analysis

We extracted the proteins in the fourth week after the success of the PD model. Proteins were extracted from tissues and cells with radioimmunoprecipitation assay lysis buffer (Beyotime, China), and the concentration of proteins was determined with bicinchoninic acid protein assay kit (Beyotime, China) according to the manufacturer’s instructions. Approximately 50 μg of protein was separated by 10% SDS-PAGE and transferred onto a polyvinylidene fluoride membrane (EMD Millipore, Bedford, MA, USA). The membranes were blocked in quick block solution (Beyotime, China) for 15 min at room temperature and subsequently incubated with primary antibodies against glyceraldehyde-3-phosphate dehydrogenase (GAPDH) (1:3,000; Proteintech, China), α-synuclein (1:1,000; Abcam, UK), tyrosine hydroxylase (TH), α-synuclein, Iba-1, IL-1β, IL-6, Nrf2 and NQO-1 (1:1,000; Proteintech), and TNF-α (Affinity, China) overnight at 4°C. The membranes were washed with tris-buffered saline with 0.1% Tween 20 and incubated with HRP-conjugated secondary antibody (Proteintech) for 1 h at room temperature. The membranes were washed again and detected using a chemiluminescence western detection system (Bio-Rad, Hercules, CA, USA). Each experiment was conducted three times, and then calculated the mean values.

### 2.7 Enzyme-linked immunosorbent assays (ELISA)

We conducted ELISA in the fourth week after the success of the PD model. The concentrations of Iba-1, IL-1β, IL-6, TNF-α, Nrf2, and NQO-1 in the tissue of the midbrain were then measured by sandwich ELISA (R&D Systems, USA) according to the manufacturer’s instructions. Each sample was measured in duplicate.

### 2.8 Statistical analyses

Statistical analyses were performed with Prism software (GraphPad 7.0, San Diego, CA, USA) and SPSS 26.0 (IBM, USA). Data are shown as the mean ± SD, and Student’s *t*-test or one-way ANOVA with Tukey’s multiple comparison test was used for the statistical analyses. *P* < 0.05 was used to indicate statistical significance.

## 3 Results

### 3.1 ILG attenuates 6-OHDA-induced neurobehavioral deficits in PD mice

**Figure 1:**
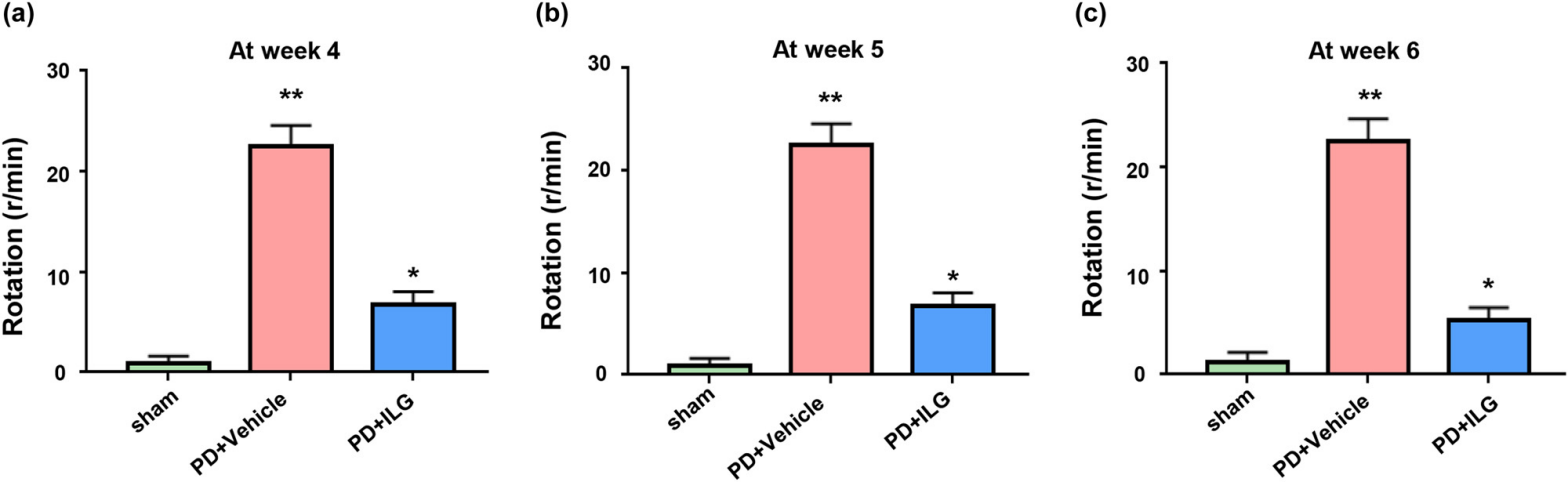
ILG attenuates neurobehavioral deficits at 4, 5, and 6 weeks after the successful establishment of PD model: (a) results of the rotation experiment in Sham, PD + Vehicle, and PD + ILG groups mice at Week 4, (b) results of the rotation experiment in the indicated groups at Week 5, and (c) results of the rotation experiment in the indicated groups at Week 6. *, *P* < 0.05; **, *P* < 0.01, *n* = 16.

To investigate the effectiveness of ILG *in vivo*, we administered ILG intraperitoneally (20 mg/kg) to 6-OHDA-induced PD mice. Then APO was injected intraperitoneally for behavioral assessment in a rotation test. The results showed a significant increase in the number of rotations in the PD + Vehicle group mice compared to the Sham group (Figure 1a–c). However, when PD mice were treated with ILG, the number of rotations decreased significantly (Figure 1). This indicates that ILG has a significant therapeutic effect on PD *in vivo*.

### 3.2 ILG has a protective effect on dopaminergic neurons and scavenges α-synuclein

**Figure 2:**
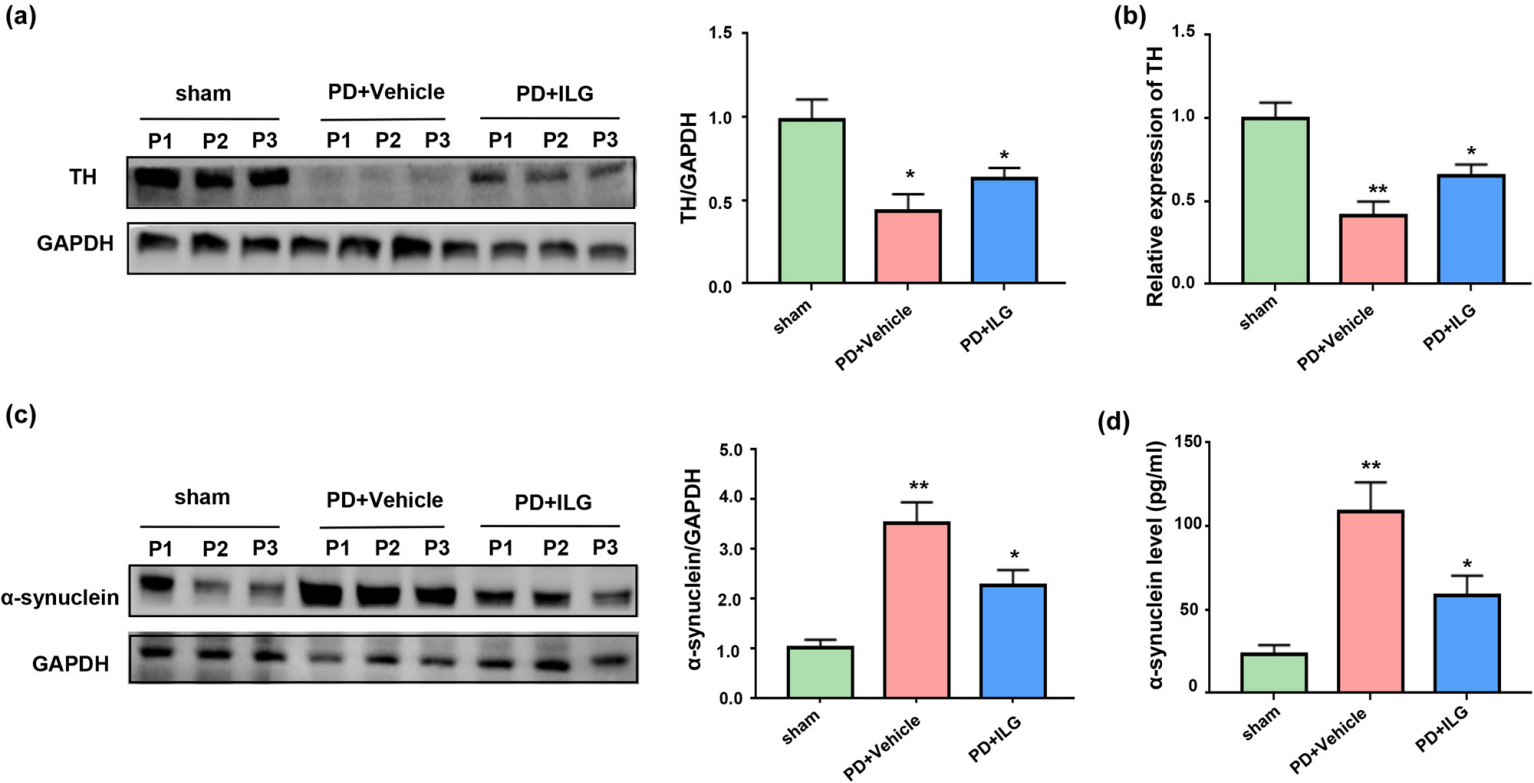
ILG has a protective effect on dopaminergic neurons and scavenges α-synuclein: (a) Western blot analysis of TH expression in the SN of midbrain tissues of the Sham, PD + Vehicle, and PD + ILG groups, (b) relative mRNA expression of TH in the SN of midbrain tissues among the three groups (qRT-PCR), (c) Western blot analysis of α-synuclein expression in the mesencephalon tissues among the three groups, and (d) levels of α-synuclein in middle brain tissues among the three groups (ELISA). *, *P* < 0.05; **, *P* < 0.01, *n* = 5.

To explore the specific mechanism of the therapeutic effect of ILG, we performed a semi-quantitative analysis of proteins for TH and α-synuclein. Our results showed that TH expression in the SN of the midbrain was significantly decreased in the PD + Vehicle group compared to the Sham group (Figure 2a and b), whereas the expression of TH was elevated in the PD + ILG group compared to the PD + Vehicle group (Figure 2a and b). This suggests that ILG has a protective effect on dopaminergic neurons. Similarly, protein expression of α-synuclein was increased in the model group after successful induction of the PD model using 6-OHDA, and decreased when ILG treatment was given (Figure 2c and d). This indicates that ILG has the effect of scavenging α-synuclein.

### 3.3 ILG alleviates neuroinflammation in PD mice

**Figure 3:**
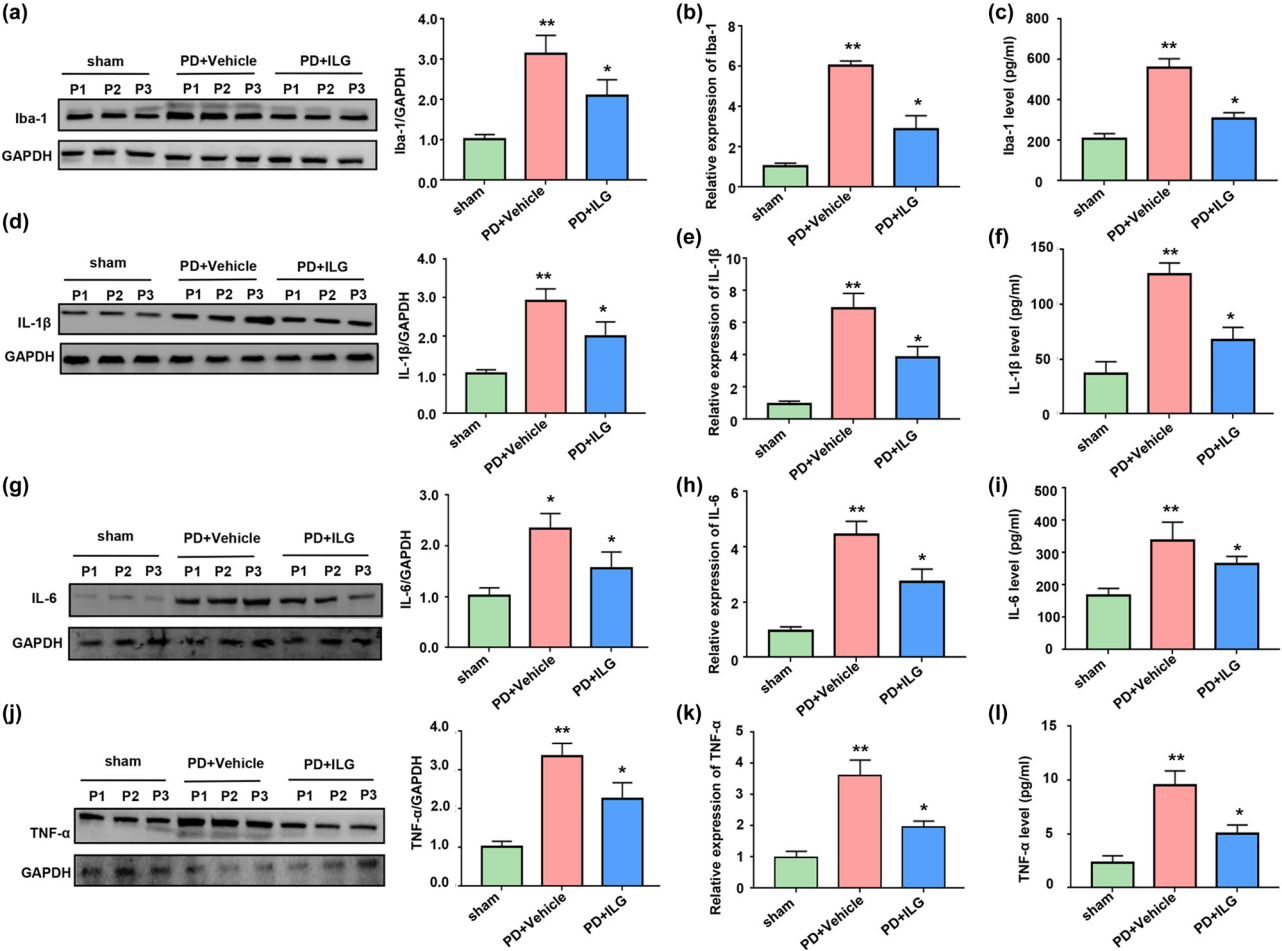
ILG alleviates neuroinflammation in PD mice: (a) protein levels of Iba-1 in the SN of midbrain tissues of the Sham, PD + Vehicle, and PD + ILG groups (Western blot), (b) mRNA levels of Iba-1 in mesencephalon tissues of the indicated groups (qRT-PCR), (c) tissue of midbrain levels of Iba-1 in the indicated groups (ELISA), (d) protein levels of IL-1β in the indicated groups (Western blot), (e) mRNA levels of IL-1β in the indicated groups (qRT-PCR), (f) tissue of midbrain levels of IL-1β in the indicated groups (ELISA), (g) protein levels of IL-6 in the indicated groups (Western blot), (h) mRNA levels of IL-6 in the indicated groups (qRT-PCR), (i) tissue of midbrain levels of IL-6 in the indicated groups (ELISA), (j) protein levels of TNF-α in the indicated groups (Western blot), (k) mRNA levels of TNF-α in the indicated groups (qRT-PCR), and (l) tissue of midbrain levels of TNF-α in the indicated groups (ELISA). *, *P* < 0.05; **, *P* < 0.01, *n* = 5.

To investigate the specific mechanism of the protective effect of ILG on dopaminergic neurons, we examined the expression of microglia and pro-inflammatory cytokines. Our results showed that ILG significantly reduced the protein expression of Iba1, a marker of microglia, suggesting that ILG significantly reduced microglial activation (Figure 3a–c). In addition, our results also showed that IL-1β, IL-6, and TNF-α were significantly higher in the PD + Vehicle group compared with the Sham group (Figure 3d, f, g, i, j and l). And these pro-inflammatory cytokines were significantly decreased after treatment with ILG (Figure 3d, f, g, i, j and l). Not only that, we also examined the relative mRNA expression levels of these pro-inflammatory cytokines, and the results remained consistent with the above results (Figure 3b, e, h and k). This indicates that ILG not only inhibits microglia activation, but also suppresses the production of pro-inflammatory cytokines. The above results strongly suggest that ILG can reduce neuroinflammation in PD.

### 3.4 ILG promotes activation of the Nrf2/NQO-1 signaling pathway

**Figure 4:**
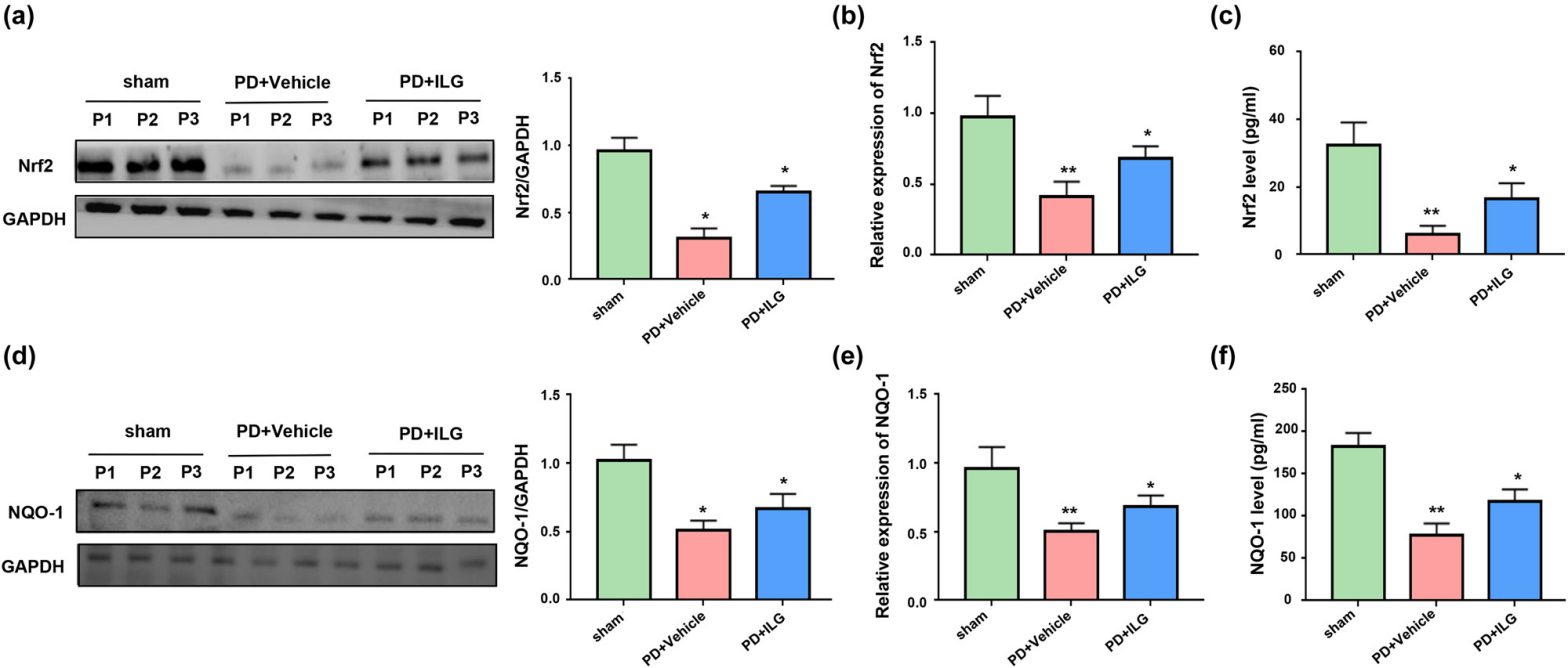
ILG promotes activation of the Nrf2/NQO-1 signaling pathway: (a) protein levels of Nrf2 in the SN of midbrain tissues of the Sham, PD + Vehicle, and PD + ILG groups (Western blot), (b) mRNA levels of Nrf2 in mesencephalon tissues of the indicated groups (qRT-PCR), (c) tissue of midbrain levels of Nrf2 in the indicated groups (ELISA), (d) protein levels of NQO-1 in the indicated groups (Western blot), (e) mRNA levels of NQO-1 in the indicated groups (qRT-PCR), and (f) tissue of midbrain levels of NQO-1 in the indicated groups (ELISA). *, *P* < 0.05; **, *P* < 0.01, *n* = 5.

To further study the deeper mechanisms of ILG to reduce neuroinflammation, we detected the relative mRNA levels and protein expression levels of Nrf2 and NQO-1. We found that the protein expression levels of Nrf2 and NQO-1 were decreased in the PD + Vehicle group compared with the Sham group (Figure 4a, c, d and f). When ILG was injected intraperitoneally, the protein expression levels of Nrf2 and NQO-1 were increased (Figure 4a, c, d and f). Moreover, the results of the gene level assay were consistent with the above results. The relative mRNA expression of Nrf2 and NQO-1 decreased in the PD model and increased when ILG treatment was given (Figure 4b–e). This indicates that ILG affected the expression of Nrf2 and NQO-1 not only at the protein level, but also at the gene level.

## 4 Discussion

PD is a chronic neurodegenerative disorder characterized by the loss of dopaminergic neurons in the SNc, accompanied by chronic neuroinflammation, oxidative stress, and α-synuclein aggregate formation. The persistent activation of neuroinflammation is an important factor exacerbating the progression of PD. ILG is thought to have a variety of biological activities including anti-inflammatory. However, ILG has not been reported in studies in animal models of PD. In the present study, we found for the first time that ILG ameliorated behavioral symptoms of PD and that this effect was achieved by promoting the Nrf2/NQO-1 signaling pathway to attenuate neuroinflammation.

In our study, we first investigated the therapeutic effect of ILG in a mouse model of PD and showed that ILG significantly improved the behavioral deficits in PD. This is the first demonstration of a positive therapeutic effect of ILG on PD *in vivo*. In addition, we found that TH expression in the midbrain SN region was significantly reduced in the 6-OHDA-induced PD model, and ILG significantly increased TH expression in the PD model, suggesting that ILG has a protective effect on dopaminergic neurons in the SN. This is consistent with the results of the *in vitro* study by Hwang and Chun [22]. However, it is worth mentioning that they only explored *in vitro* and did not validate the effect of ILG *in vivo* and also did not validate the behavior of PD mice after ILG injection. The present study validated the effectiveness of ILG in PD *in vivo* and it is an effective supplement to this study *in vitro*. Meanwhile, we also found that ILG could reduce the expression of α-synuclein, which suggests that ILG could play a role in scavenging α-synuclein *in vivo*. It is well known that the abnormal aggregation of α-synuclein is one of the important mechanisms in the pathogenesis of PD. The above results suggest that ILG can have a protective effect on dopaminergic neurons *in vivo* by scavenging α-synuclein.

Moreover, we found that ILG downregulated the expression of microglia-specific marker Iba1, suggesting that ILG inhibited microglial activation. ILG also downregulated the expression of various pro-inflammatory cytokines such as IL-1β, IL-6, and TNF-α. These results reveal that ILG attenuates neuroinflammation in the SN of PD mice. This is similar to the findings of Liu et al. [18], who also demonstrated that ILG attenuated neuroinflammation, but their study was mainly explored in a TBI model. Our study is the first to find that ILG reduces neuroinflammation in a PD model.

To further investigate the mechanism by which ILG attenuates neuroinflammation, we probed the signaling pathways at the genetic and molecular levels. We found that Nrf2 and NQO-1 expression was upregulated in the 6-OHDA-induced PD model, while Nrf2 and NQO-1 expression was downregulated when treated with ILG. This is similar to the findings of Zeng et al. [17] and Zhang et al. [19], whose results suggest that ILG exerted neuroprotective effects by promoting the Nrf2/ARE signaling pathway to attenuate neurobehavioral deficits after intracerebral hemorrhage. Furthermore, Cheng et al. [23] found that ghrelin attenuated inflammasome expression by promoting the expression of Nrf2/ARE signaling. Most importantly, Zeng et al. [17] demonstrated that ILG can downregulate the expression of inflammasome after intracerebral hemorrhage in rats by promoting the Nrf2/ARE signaling pathway. This suggests that Nrf2-associated signaling pathways are closely related to neuroinflammation. Most studies have shown that Nrf2/ARE pathway has become an important target for the prevention and treatment of oxidative stress-related neurodegenerative diseases. The small molecular inducers of Nrf2/ARE pathway include l-sulforaphane in broccoli and ILG in licorice, which show good protective effect on mitochondrial function in the model of oxidative stress and neurodegenerative diseases, and represent a new way to prevent and treat aging-related neurodegenerative diseases. However, unfortunately, these studies are based on the mechanism of oxidative stress, and our study found a link between Nrf2-related mechanisms and neuroinflammation, and proved the role of ILG on PD *in vivo* [20,24].

In this study, we first found the positive therapeutic effect of ILG *in vivo* in a PD mouse model, and confirmed that ILG reduced neuroinflammation in a PD model and validated the effect of ILG on the Nrf2/NQO-1 signaling pathway in a PD model. In summary, our study suggests that ILG may be a potential therapeutic agent for the clinical treatment of PD in the future and deserves further development and investigation.

## 5 Conclusions

Taken together, ILG significantly attenuated neurological dysfunction in experimental PD, and the mechanisms may be through activation of the Nrf2/NQO-1 signaling pathway to alleviate neuroinflammation. Furthermore, our findings provide a new therapeutic strategy for PD.
